# Development and validation of a predictive model for severe radiation-induced esophagitis in lung cancer patients undergoing moderate hypofractionated radiotherapy

**DOI:** 10.3389/fonc.2025.1656907

**Published:** 2025-10-03

**Authors:** Anqi Zhang, Mengjie Quan, WeiQian Li, Jing Cao, Yuee Liu, Bo Zhu, Xiaocang Ren, Yueliang Qin, Qiang Lin

**Affiliations:** ^1^ Oncology Department, Huabei Petroleum Administration Bureau General Hospital, Hebei Medical University, Renqiu, Hebei, China; ^2^ Anesthesiology Department, Huabei Petroleum Administration Bureau General Hospital, Hebei Medical University, Renqiu, Hebei, China

**Keywords:** radiation-induced esophagitis, lung cancer, moderate hypofractionated radiotherapy, predictive model, elastic net regression

## Abstract

**Background and objectives:**

Moderate hypofractionated radiotherapy (MHRT) is an important treatment modality for lung cancer, offering shorter courses and improved local control, yet it also markedly increases the risk of severe radiation-induced esophagitis (RIE; grade ≥3). Severe RIE compromises quality of life and adherence to therapy and may necessitate interruption of radiotherapy. This study aimed to develop a prediction model based on clinical and dosimetric factors to identify high-risk patients receiving MHRT and to facilitate individualized treatment strategies.

**Methods:**

Lung cancer patients receiving moderate hypofractionated radiotherapy were included, with the endpoint defined as grade ≥3 radiation-induced esophagitis. Baseline characteristics were summarized using non-missing data only. During model development, in each outer bootstrap training set, candidate variables underwent single-rule imputation (median for continuous variables, mode for categorical variables) and standardization, followed by variable selection via elastic-net regression and model building with Firth-penalized logistic regression; the outcome itself was not imputed. Fully nested bootstrap validation (B=1000) was performed to assess internal robustness, with optimism-corrected performance metrics and 95% confidence intervals reported. Discrimination was evaluated using ROC curves and AUC, calibration by calibration plots and the Hosmer–Lemeshow test, and clinical utility through decision curve analysis (DCA). The optimal threshold was determined by the Youden index, with the corresponding confusion matrix presented. Finally, a nomogram was constructed to facilitate clinical visualization and application.

**Results:**

A total of 105 patients were included; the incidence of grade ≥3 RIE was 16.2% (17/105). Five predictors entered the final model via elastic-net selection: mean gross tumor volume (mean GTV), V5, D2cc, circumferential 2.6-Gy irradiated length, and circumferential 3.0-Gy irradiated length. The Firth-penalized logistic model showed good apparent performance: AUC=0.771, Brier score = 0.114, calibration slope = 1.16, and calibration intercept = 0.13. After optimism correction by fully nested bootstrap (B=1,000), discrimination decreased to AUC=0.608 (95% CI, 0.464–0.761) with a corresponding Brier score of 0.176 (95% CI, 0.114–0.247). The Hosmer–Lemeshow test yielded χ² = 7.84, p = 0.449, indicating acceptable overall fit. The Youden-index–derived optimal cutoff was 0.130, stratifying patients into high-risk (predicted probability ≥ 0.13) and lower-risk (< 0.13) groups. DCA demonstrated positive net benefit over “treat-all” and “treat-none” strategies across threshold probabilities of 0–0.8. Optimism-corrected calibration parameters were unstable, likely reflecting the limited number of events; these results should be interpreted with caution.

**Conclusion:**

Using elastic-net feature selection and Firth logistic regression, we developed a model to predict severe (grade ≥3) RIE in lung cancer patients undergoing MHRT. The model exhibited moderate discriminatory ability with generally acceptable calibration, enables risk stratification and identification of high-risk patients, and is presented as a nomogram to support clinical application. It holds promise for guiding individualized radiotherapy decisions and the prevention of treatment-related complications.

## 1 Introduction

Lung cancer remains one of the most prevalent and lethal malignancies worldwide (1, 2). Radiotherapy plays a pivotal role in the management of lung cancer across various stages, including early, locally advanced, and certain metastatic cases. Although concurrent chemoradiotherapy has become the standard treatment for patients with locally advanced non-small cell lung cancer (LA-NSCLC), its high toxicity profile has led to the continued widespread use of sequential chemoradiotherapy in clinical settings (3–5). In recent years, moderate hypofractionated radiotherapy has gained clinical traction due to its ability to shorten overall treatment duration while enhancing the biologically effective dose (BED), demonstrating promising therapeutic efficacy (6–9). However, treatment-related toxicities, particularly acute radiation-induced esophagitis (RIE), remain major complications that hinder the smooth progression of therapy (10, 11).

Radiation-induced esophagitis is among the most common toxic effects of thoracic radiotherapy, with an incidence as high as 30–50% in lung cancer patients receiving concurrent chemoradiotherapy. Grade ≥3 RIE, in particular, significantly impairs quality of life, nutritional status, and treatment compliance, potentially leading to therapy interruption or dose reduction, ultimately compromising therapeutic efficacy (12, 13). The challenge is even more pronounced under moderate hypofractionated regimens, where higher per-fraction doses subject the esophagus to increased stress, underscoring the urgency of identifying high-risk patients early.

Currently, most RIE risk prediction models focus on conventional fractionation (1.8–2.0 Gy) and SBRT/SABR, with limited research specifically addressing moderate hypofractionated protocols. Moreover, many existing models rely solely on univariate analysis or traditional logistic regression without rigorous feature selection or stability validation. Thus, the development of an accurate, robust, and generalizable model for predicting RIE under moderate hypofractionated radiotherapy is imperative for guiding individualized treatment decisions.

Drawing on real-world clinical data, this study employed elastic-net regression for feature selection and Firth-penalized logistic regression to construct a predictive model, thereby mitigating estimation bias associated with small samples and rare events. Internal validation and optimism correction were performed via a fully nested bootstrap procedure, and model performance was evaluated multidimensionally through ROC analysis, calibration plots, the Hosmer–Lemeshow test, and DCA. The Youden index was used to determine the optimal classification threshold, and a nomogram was developed to enable intuitive clinical application. Our goal was to create an accurate, robust, and clinically generalizable tool for the early identification of patients at high risk of RIE following MHRT, thereby providing a rigorous foundation for individualized radiotherapy optimization and the prevention of treatment-related complications.

## 2 Materials and methods

### 2.1 Study population and data source

This study enrolled 105 lung cancer patients who received moderate hypofractionated thoracic radiotherapy at Huabei Petroleum Administration Bureau General Hospital between January 2017 and December 2022. Inclusion criteria were: ①histologically confirmed lung cancer; ② moderate hypofractionation regimen (3.0 Gy/fraction, 5 fractions/week, total dose 45–60 Gy); ③ availability of complete clinical and dosimetric data; and ④ absence of pre-existing esophageal disease. Exclusion criteria included: ① concurrent surgical intervention; ② treatment discontinuation unrelated to radiotherapy toxicity; and ③ presence of severe comorbidities compromising prognostic evaluation.

All patients provided informed consent, and the study protocol was approved by the institutional ethics committee.

### 2.2 Grading of radiation-induced esophagitis

Patients underwent weekly clinical evaluations during the course of moderate hypofractionated radiotherapy to monitor adverse events, including esophagitis, followed by radiographic assessments. Grading was performed in accordance with the National Cancer Institute’s Common Terminology Criteria for Adverse Events, version 5.0 (CTCAE 5.0). Radiation-induced esophagitis (RIE) was stratified into two groups: grades 0–2 constituted the low-risk group, while grade ≥3, representing clinically significant symptoms, defined the high-risk group. This dichotomization served as the binary outcome variable for the study (y = 0 for RIE ≤2; y = 1 for RIE ≥3).

### 2.3 Variable definition and extraction

Scope and cohort: To develop an actionable, planning-stage prediction model, we initially considered 32 dosimetric and geometric variables directly tied to the radiotherapy plan. Several clinical characteristics (e.g., age, stage, comorbidities) were recorded for baseline description only and were not included in model building.

Structure and data sources: The esophagus was contoured from the cricoid cartilage to the gastroesophageal junction; an Esophagus–GTV structure was generated by subtracting the GTV overlap from the esophageal contour to quantify extratumoral esophageal dose. All dosimetric parameters were automatically derived from dose–volume histograms (DVHs) exported by the treatment planning system (TPS). Doses were reported in Gy (with cGy converted to Gy before analysis); volume fractions as percentages; and lengths in centimeters.

Candidate variables: Conventional DVH metrics included Esophagus–GTV mean dose (Dmean) and whole-esophagus Dmean; point maximum dose (Dmax, expressed as D0.03 cm³ or D2%); V5–V60 (fractional volume receiving ≥x Gy); and D1/2/3 cm³ (minimum dose to the hottest 1/2/3 cm³). Circumferential metrics comprised the maximum per-fraction circumferential dose and the cumulative esophageal length receiving ≥2.0–3.0 Gy per fraction. Clinical variables encompassed smoking history, Karnofsky Performance Status (KPS; ≥80 *vs <*80), prior chemotherapy (yes/no), the number of involved nodal stations (IASLC classification), and involvement of stations 4L and 7 (yes/no).

### 2.4 Statistical analysis

Baseline characteristics were stratified by outcome (<G3 *vs* ≥G3) and expressed as mean ± standard deviation, median [IQR], or n (%), with percentages calculated using non-missing samples as the denominator. Continuous variables were first assessed for normality using the Shapiro–Wilk test, and homogeneity of variance was evaluated with Levene’s test. When both assumptions were satisfied, comparisons were performed with the independent-samples t-test (applying Welch’s correction when necessary); otherwise, the Mann–Whitney U test was employed. Categorical variables were compared using the χ² test, and when any expected cell count was <5, Fisher’s exact test was adopted. All statistical tests were two-sided, with a significance threshold of α = 0.05. Reported p-values were used solely to describe intergroup differences and were not intended for variable selection or causal inference. Missing data were handled on a per-variable basis using available cases without imputation.

This single-center retrospective cohort adhered to TRIPOD. Given the limited number of events (≥G3, 16.2%), we did not split the data into training and validation sets; instead, internal validation employed a fully nested bootstrap (B=1,000) to obtain robust performance estimates and optimism correction.

Within the framework of fully nested bootstrap, each outer training set first underwent single-rule imputation of candidate predictors (median for continuous variables, mode for categorical variables), followed by z-score standardization. Feature selection was then performed using elastic-net regression (α = 0.5, with λ determined by tenfold cross-validation), after which the final model was constructed with Firth-penalized logistic regression (logistf package). Imputation and standardization parameters estimated from the training set were applied solely to the corresponding validation set to prevent information leakage. Variable selection was re-executed in every iteration to mitigate selection bias. Model effect sizes were reported as regression coefficients (β), odds ratios (OR), and their 95% confidence intervals (CI).

Discrimination was assessed via ROC and the area under the curve (AUC). The optimal cutoff was determined by the Youden index, with sensitivity, specificity, positive predictive value (PPV), negative predictive value (NPV), overall accuracy, and a confusion matrix subsequently calculated. Calibration was examined with bootstrap-based (B=1,000) calibration plots and by estimating the calibration slope and intercept; the Hosmer–Lemeshow test (ResourceSelection package, g = 10) provided an additional assessment of goodness-of-fit, with P > 0.05 indicating acceptable fit.

Clinical utility was evaluated using DCA, comparing net benefit against “treat-all” and “treat-none” strategies across threshold probabilities of 0–0.8. Finally, a nomogram was constructed to visualize individualized risk and support clinical stratification and decision-making.

All statistical analyses were conducted in R (version 4.2.3).

## 3 Results

### 3.1 Variable selection via elastic net regression

A total of 105 patients were enrolled, with an incidence of ≥G3 radiation-induced esophagitis of 16.2% (17/105). Within the overall cohort, 73.3% (77/105) were male, with a mean age of 65.0 ± 9.8 years and a median of 66.0 years [Q1–Q3: 58.0–72.0]. A history of smoking was present in 39.0% (41/105). The distribution of stage III/IV disease was 34.3% and 65.7%, respectively. KPS ≥80 was observed in 76.0% (79/104). NSCLC accounted for 82.9% (87/105), and prior chemotherapy was reported in 82.9% (87/105).

Comparisons between groups revealed no statistically significant differences in sex (P=0.550), age (P=0.556), smoking history (P=0.729), tumor stage (P=0.513), KPS (P=1.000), histological type (P=0.294), or prior chemotherapy (P=1.000) (**Table 1**), indicating broadly comparable baseline characteristics. The only significant distinction was in comorbidity rates, which were lower in the ≥G3 group (41.2% *vs*. 72.7%, P=0.011). This unexpected directionality suggests potential influences of limited sample size, treatment selection, and unmeasured confounders; definitive conclusions should rely on the results of multivariable modeling.

**Table 1:** Baseline characteristics stratified by RIE severity (<G3 *vs* ≥G3).

Overall (N=105)	Radiation-induced Esophagitis(RIE)		P
	<G3	≥G3	
			0.5501
77 (73.3%)	63 (71.6%)	14 (82.4%)	
28 (26.7%)	25 (28.4%)	3 (17.6%)	
65.0 ± 9.8; 66.0 [58.0, 72.0]	65.2 ± 10.2; 66.0 [57.8, 73.0]	63.9 ± 7.9; 64.0 [61.0, 70.0]	0.5561
56 (53.3%)	48 (54.5%)	8 (47.1%)	
49 (46.7%)	40 (45.5%)	9 (52.9%)	
			0.7290
64 (61.0%)	53 (60.2%)	11 (64.7%)	
41 (39.0%)	35 (39.8%)	6 (35.3%)	
			0.5132
36 (34.3%)	29 (33.0%)	7 (41.2%)	
69 (65.7%)	59 (67.0%)	10 (58.8%)	
			0.0109
34 (32.4%)	24 (27.3%)	10 (58.8%)	
71 (67.6%)	64 (72.7%)	7 (41.2%)	
			1.0000
79 (76.0%)	66 (75.0%)	13 (76.5%)	
25 (24.0%)	21 (23.9%)	4 (23.5%)	
			0.2940
87 (82.9%)	71 (80.7%)	16 (94.1%)	
18 (17.1%)	17 (19.3%)	1 (5.9%)	
			1.0000
18 (17.1%)	15 (17.0%)	3 (17.6%)	
87 (82.9%)	73 (83.0%)	14 (82.4%)	

Continuous variables are presented as mean ± standard deviation, accompanied by median [interquartile range], while categorical variables are expressed as n (%). P-values, derived from t-tests or Wilcoxon rank-sum tests for continuous variables and χ² or Fisher’s exact tests for categorical variables, are reported for descriptive comparisons.

### 3.2 Variable selection and final model

Among the 32 plan-related candidate metrics, elastic-net regression (α = 0.5) retained five predictors for model entry: mean GTV, V5, D2cc (minimum dose to the hottest 2 cm³), the circumferential length receiving ≥2.6 Gy per fraction, and the circumferential length receiving ≥3.0 Gy per fraction. Multivariable estimation was then performed using Firth-penalized logistic regression. All continuous covariates were z-standardized prior to modeling; consequently, each odds ratio (OR) reflects the relative risk associated with a one–standard deviation increase in the predictor.

**Figure 1:**
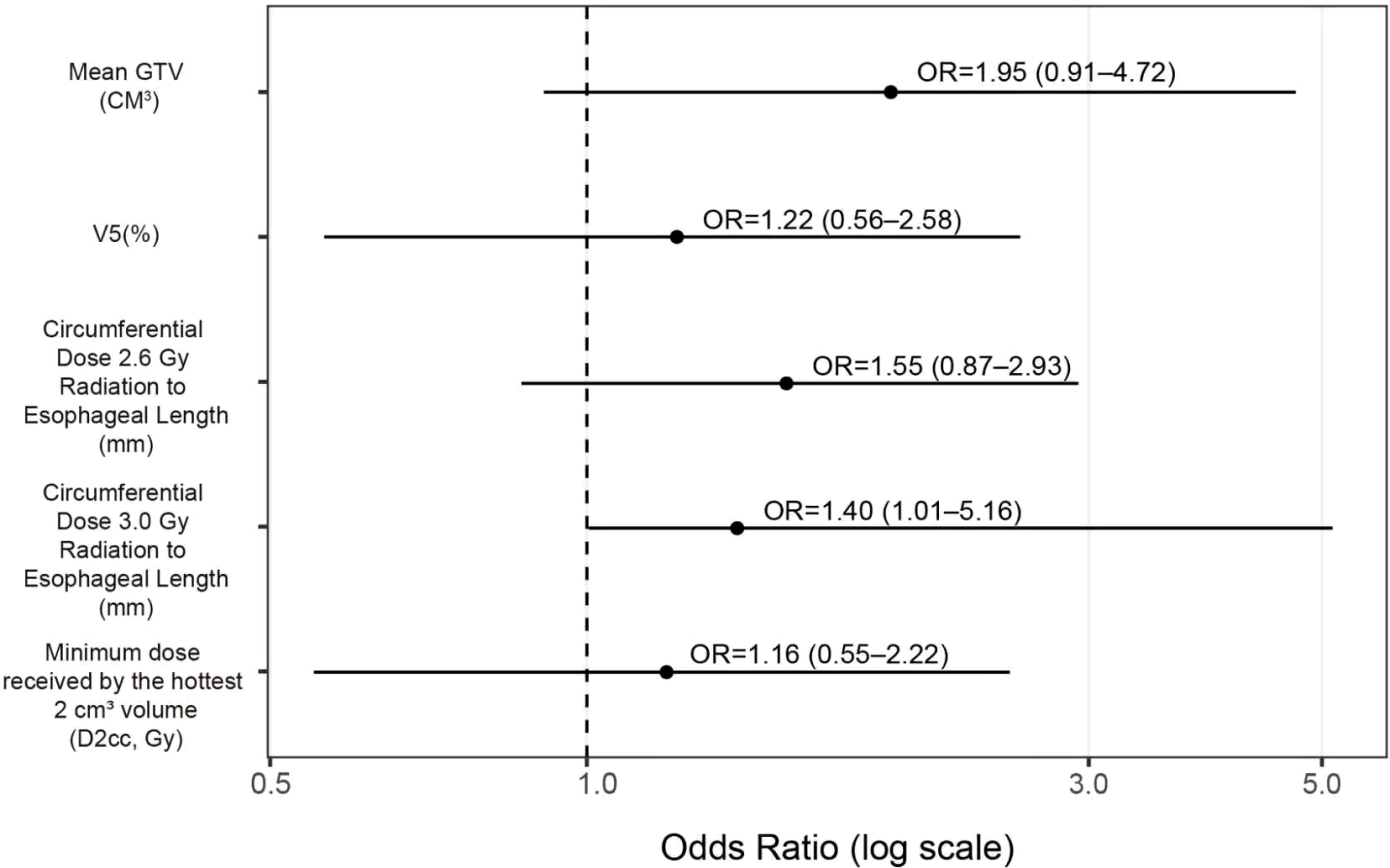
Forest plot for predicting ≥G3 RIE (Firth-penalized logistic regression). Black dots denote OR point estimates, horizontal lines the 95% CIs; the vertical dashed line marks OR=1 (no effect); the x-axis is on a logarithmic scale. Because continuous predictors were z-standardized before model entry, ORs represent the change in risk per one–standard deviation increase.

The forest plot indicates that all five variables are positively associated with the risk of ≥G3 RIE, with the circumferential ≥3.0-Gy length emerging as an independent correlate (OR=1.40, 95% CI 1.01–5.16). The remaining predictors did not reach statistical significance but showed concordant directions, suggesting an upward risk trend (see **Figure 1**): mean GTV, OR 1.95 (95% CI 0.91–4.72); V5, OR 1.22 (0.56–2.58); circumferential length ≥2.6 Gy/fx, OR 1.55 (0.87–2.93); D2cc, OR 1.16 (0.55–2.22).

### 3.3 Model discrimination

**Figure 2:**
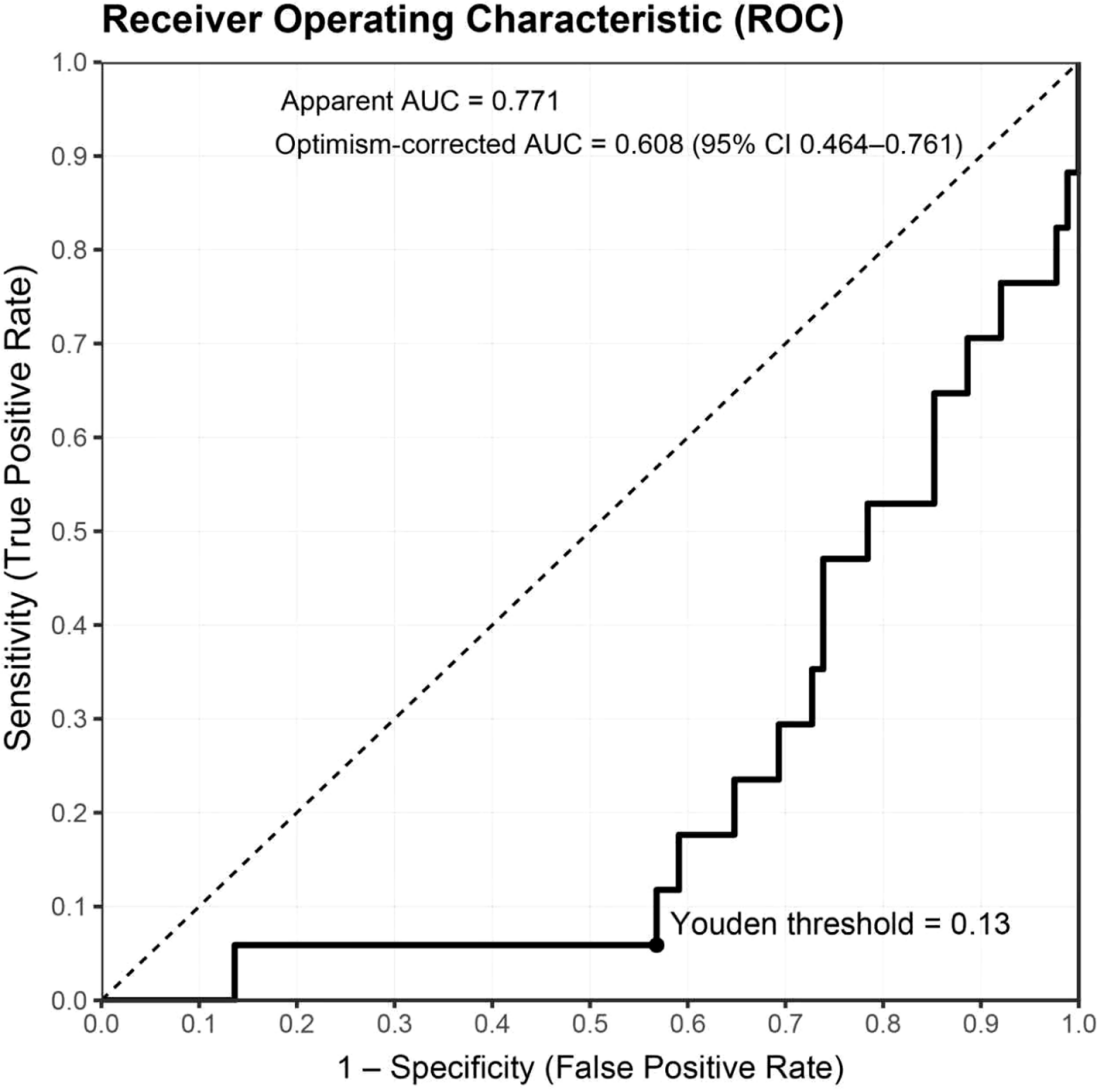
ROC curve and threshold performance for predicting ≥G3 RIE. The bold black line depicts the apparent ROC (AUC=0.771); the dashed diagonal represents chance. After fully nested bootstrap correction, AUC=0.608 (95% CI 0.464–0.761). The solid dot marks the optimal cutoff based on the maximal Youden index (0.1299 ≈ 0.13). The x- and y-axes denote 1 − specificity (false-positive rate) and sensitivity (true-positive rate), respectively. Sample size N=105; events = 17 (16.2%). Predicted probabilities derive from the full-sample model using elastic-net selection followed by Firth-penalized logistic regression; continuous predictors were z-standardized prior to modeling.

Under the apparent fit, the model demonstrated good discrimination (AUC=0.771) and a low probabilistic error (Brier = 0.114). Following internal validation with a fully nested bootstrap (B=1,000) and optimism correction, discrimination declined to AUC=0.608 (95% CI 0.464–0.761), with a corresponding Brier score of 0.176 (95% CI 0.114–0.247), indicating some overfitting in the context of few events yet an overall moderate discriminative capacity. For clinical interpretability, summary metrics are reported in **Table 2**, including both the apparent AUC and the optimism-corrected AUC derived from the fully nested bootstrap (B=1,000). The optimal threshold determined by the maximal Youden index was 0.130; at this cutoff, sensitivity was 94.1%, specificity 56.8%, accuracy 62.9%, PPV 29.6%, and NPV 98.0%. Given the low event rate of ≥G3 in this cohort, the model offers strong rule-out performance (high NPV), supporting early alerting and intensified follow-up for patients at elevated risk. The ROC curve is shown in **Figure 2**.

**Table 2:** Discriminative performance and classification metrics at the Youden-derived cutoff.

Metric	Value
Area Under Curve (AUC)	0.771
Optimism-corrected AUC	0.608
Optimal Cutoff Value	0.130
Sensitivity (%)	94.1
Specificity (%)	56.8
Accuracy (%)	62.9
Positive Predictive Value (PPV) (%)	29.6
Negative Predictive Value (NPV) (%)	98.0

AUC (apparent) denotes the unadjusted value; the optimism-corrected AUC was obtained via a fully nested bootstrap (B=1,000). The optimal cutoff was defined by the maximal Youden index; sensitivity, specificity, accuracy, PPV, and NPV were computed at this threshold on the full dataset. PPV/NPV are prevalence-dependent; the ≥G3 event rate in this cohort was 16.2%.

### 3.4 Model calibration

**Figure 3:**
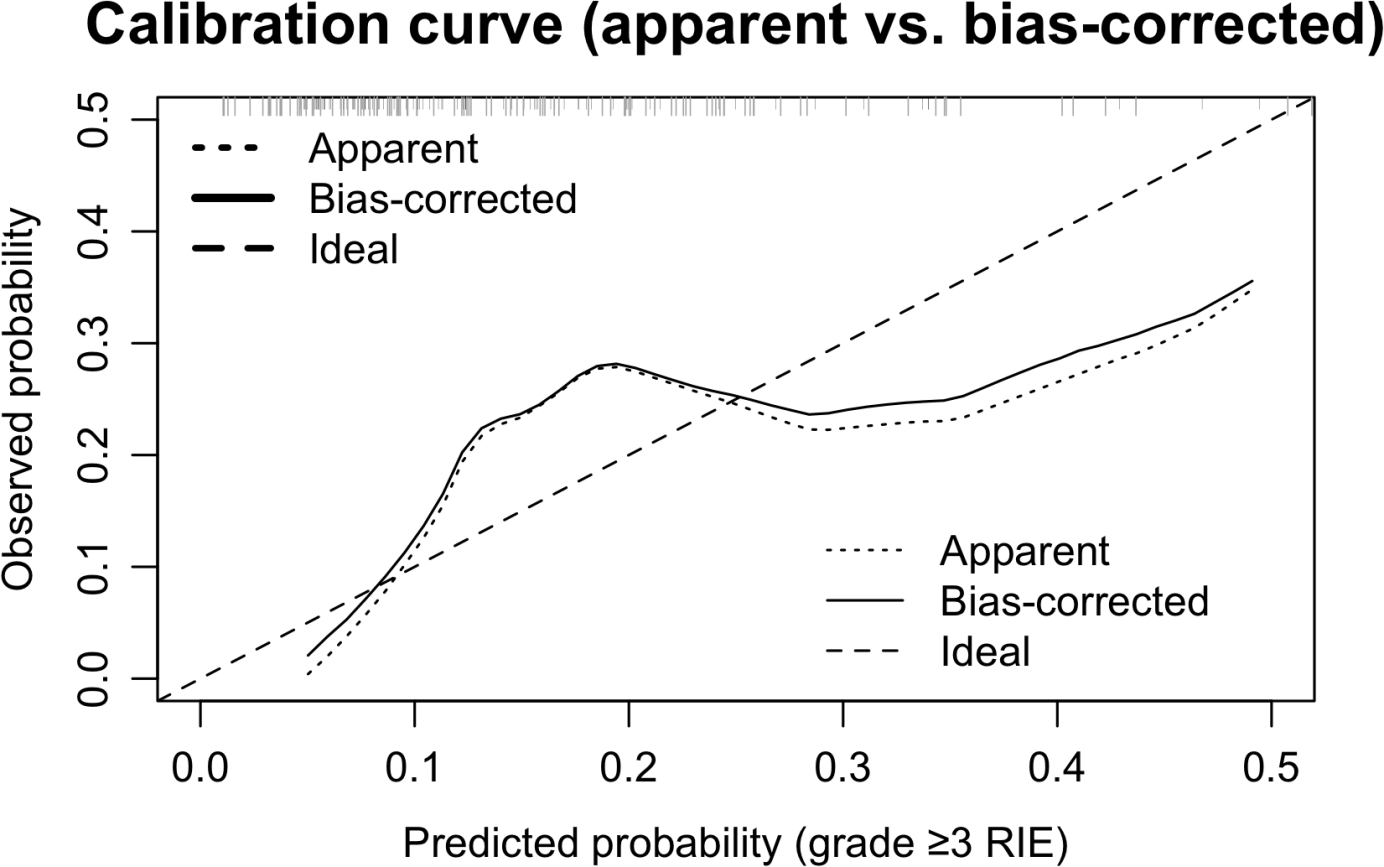
Calibration curves (apparent *vs* bias-corrected). The dotted line denotes apparent calibration, the solid line the bootstrap bias-corrected curve (B=1000), and the dashed line the ideal y = x; rug marks above the axis indicate the distribution of predicted probabilities. Alignment with the ideal line in the low–moderate range (~0.10–0.25) supports overall acceptable calibration. Apparent slope/intercept were 1.16/0.13; Hosmer–Lemeshow χ² = 7.84, p = 0.449.

Apparent calibration for the full sample yielded a slope of 1.16 and an intercept of 0.13, with the Hosmer–Lemeshow test showing χ² = 7.84, p = 0.449, indicating an overall acceptable fit. As illustrated in the calibration curve (**Figure 3**), the model closely aligned with the ideal line within the low-to-moderate predicted probability range (approximately 0.10–0.25), while a slight overestimation of risk emerged when probabilities exceeded ~0.25. After optimism correction using fully nested bootstrap resampling (B=1000), the point estimates of calibration slope and intercept attenuated, with substantially widened intervals, reflecting greater parameter uncertainty under the constraint of limited event numbers. Taken together, the model demonstrates acceptable calibration within the clinically most relevant probability range, though lightweight recalibration (intercept/slope adjustment) in external populations will be necessary to further enhance its generalizability.

### 3.5 Decision curve analysis

**Figure 4:**
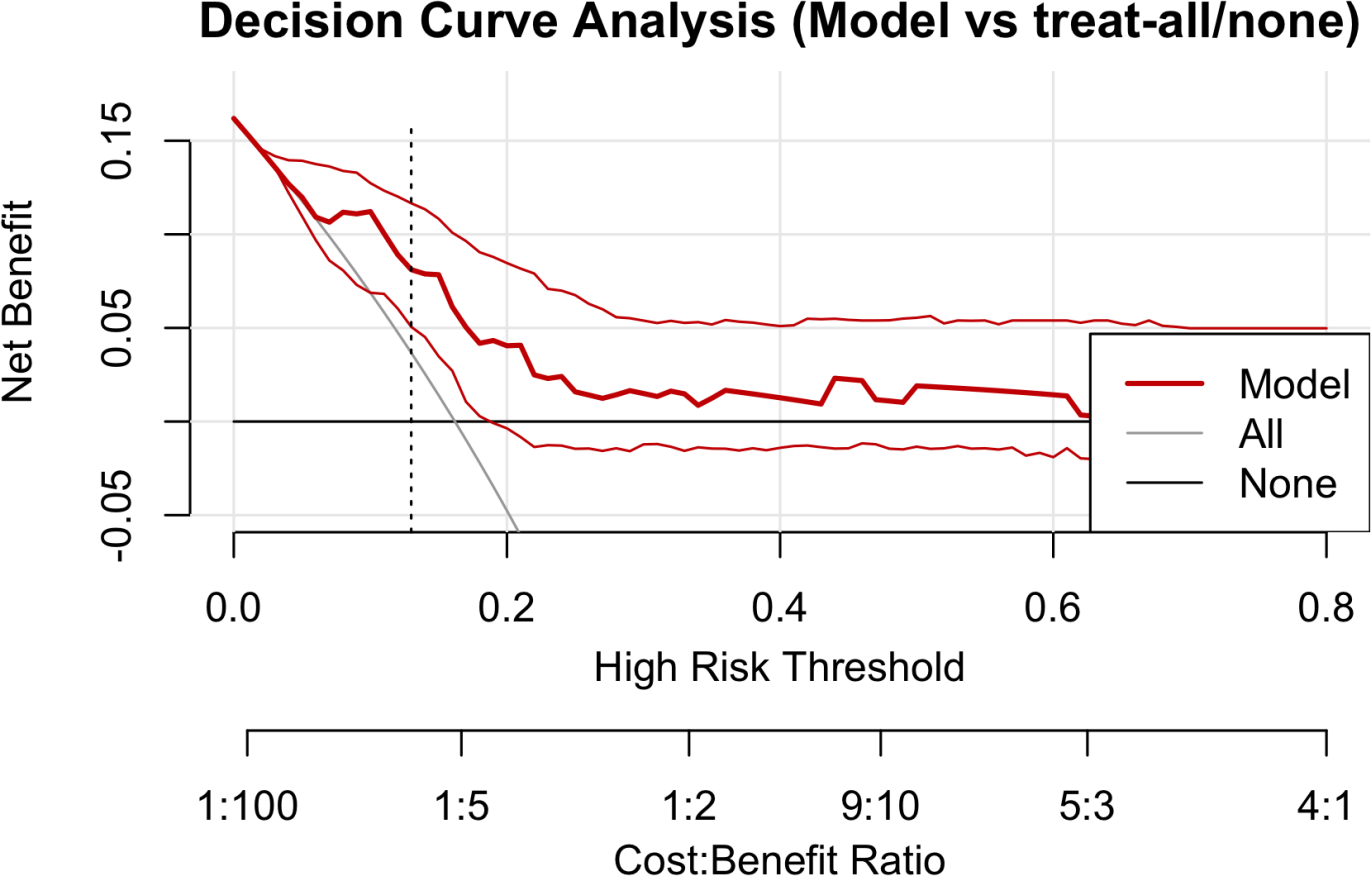
Decision curve analysis. The red solid line denotes model net benefit; the gray and black lines represent “treat-all” and “treat-none,” respectively. The vertical dashed line marks the Youden-based optimal threshold (pt ≈ 0.13). Net benefit is positive and exceeds both extreme strategies between 0.05 and 0.35, tapering toward the baselines at higher thresholds.

Using the rmda framework, DCA was performed across threshold probabilities of 0–0.80 with 500 bootstrap resamples (**Figure 4**). Within commonly used clinical thresholds, the model’s curve lay consistently above the “treat-all” and “treat-none” baselines: between ~0.05 and 0.35, the model conferred sustained positive net benefit, indicating that model-guided intervention outperforms either extreme strategy. As thresholds increased further, net benefit gradually approached the baselines. At the ROC-derived optimal threshold of pt ≈ 0.13 (maximal Youden index), the net benefit was ~0.03–0.04, equivalent to ≈3–4 net true positives per 100 patients—avoiding unnecessary interventions without increasing missed cases. Collectively, the model yields tangible clinical gain across low-to-moderate thresholds, supporting its use for early alerting and intensified follow-up in high-risk patients.

### 3.6 Classification performance and risk stratification at the optimal threshold

**Figure 5:**
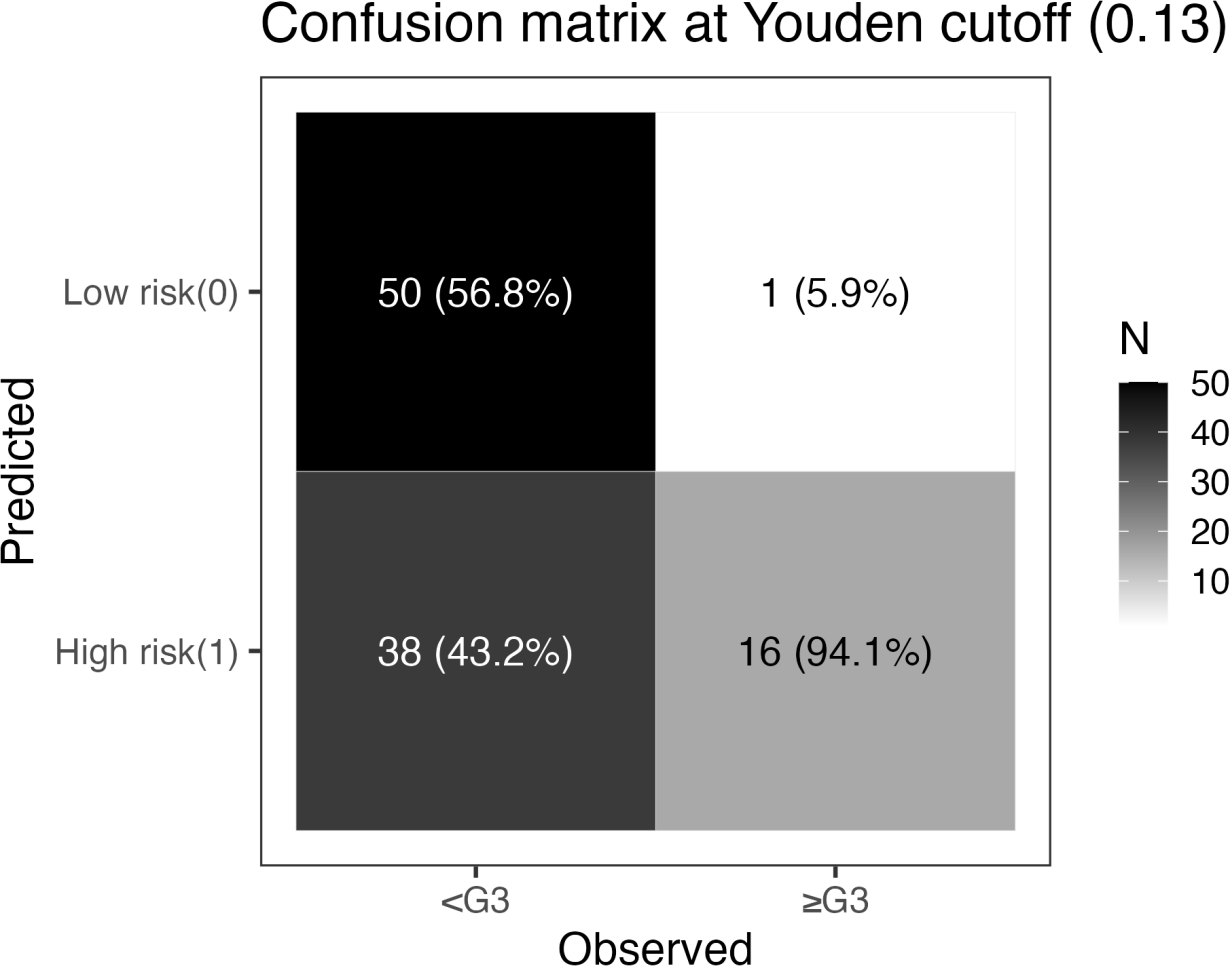
Confusion matrix at the Youden threshold (pt = 0.13). Rows indicate model predictions (top: low risk [0]; bottom: high risk [1]); columns indicate observed outcomes (left: no ≥G3 RIE; right: ≥G3 RIE). Each cell shows counts, with column percentages in parentheses (proportion within the observed-outcome column): TN=50 (56.8%), FN=1 (5.9%), FP=38 (43.2%), TP=16 (94.1%). Derived metrics: sensitivity 94.1%, specificity 56.8%, accuracy 62.9%, PPV 29.6%, NPV 98.0%. The grayscale bar on the right encodes count magnitude.

Using the ROC-derived maximal Youden index, a threshold of 0.13 was selected to stratify patients into high-risk (predicted probability ≥0.13) and low-risk (<0.13) groups. At this threshold, the confusion matrix (**Figure 5**) was: TP=16, FN=1, TN=50, FP=38. The corresponding classification metrics were: sensitivity 94.1% (16/17), specificity 56.8% (50/88), accuracy 62.9% (66/105), PPV 29.6% (16/54), and NPV 98.0% (50/51). After stratification, the incidence of ≥G3 RIE was 29.6% in the high-risk group (16/54) versus 2.0% in the low-risk group (1/51), a marked separation indicating strong screening and rule-out performance; missed cases were rare (only 1), aligning with the positive net benefit observed in DCA across thresholds of 0.05–0.35. Taken together, the 0.13 cutoff supports early identification of high-risk individuals with intensified monitoring/intervention, while enabling treatment escalation and streamlined follow-up for low-risk patients; nonetheless, given the limited number of events, this threshold warrants external validation and potential recalibration.

### 3.7 Variable selection stability (bootstrap selection frequency)

**Figure 6:**
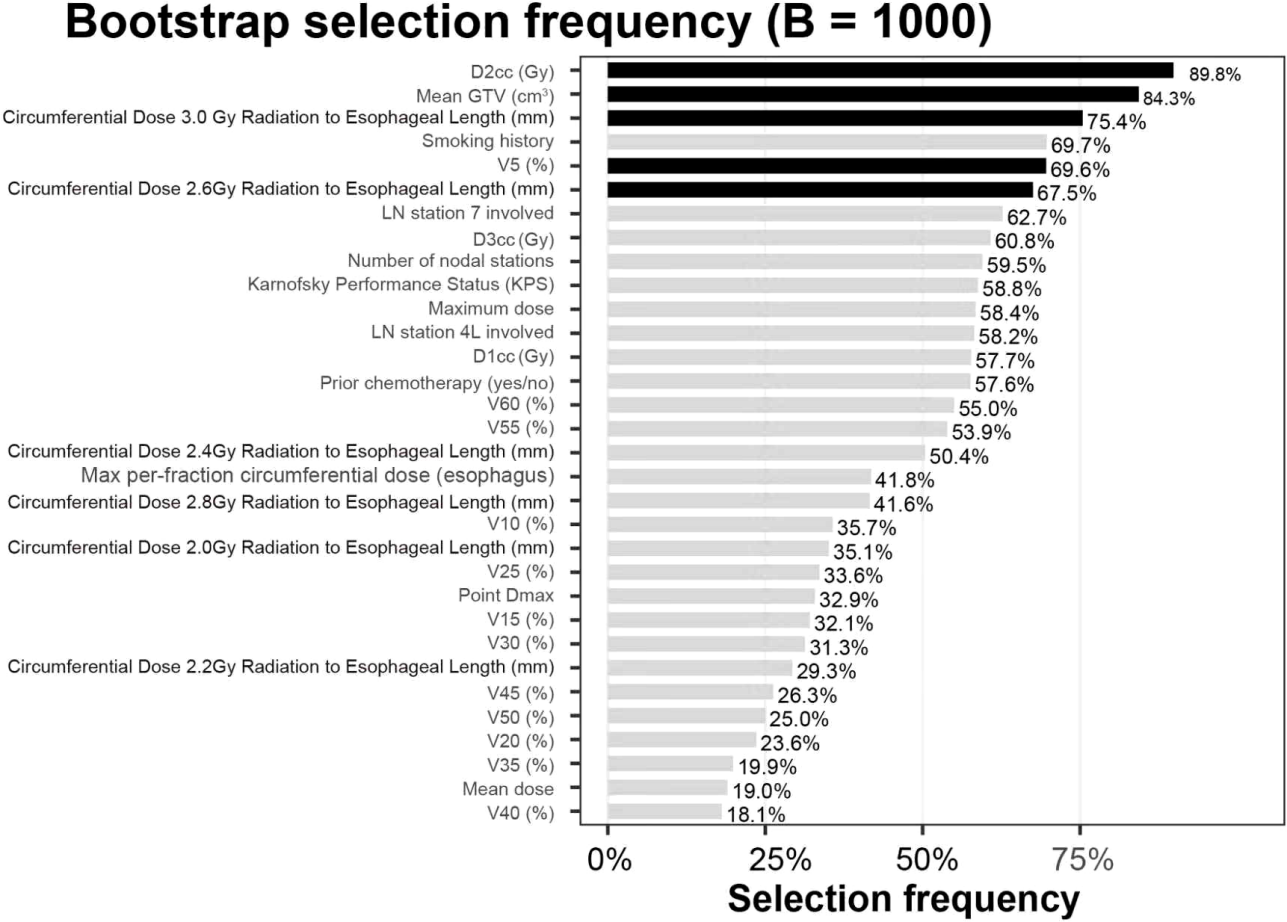
Variable selection frequencies (B=1,000). The bar chart depicts, for each candidate, the proportion selected into the model across 1,000 fully nested bootstrap iterations (selection frequency, %). Black bars denote the five retained predictors: mean GTV (cm³), V5 (%), circumferential length ≥2.6 Gy/fx (mm), circumferential length ≥3.0 Gy/fx (mm), and D2cc³ (Gy). Gray bars indicate non-selected candidates. Values above bars represent selection frequency percentages.

Using a fully nested bootstrap (B=1,000) to assess the stability of variable selection, we achieved selection_success = 1000/1000 valid iterations. For each candidate variable, we tallied the number and proportion of times it was included in the final model across resamples (selection frequency) and visualized the results with a bar plot. Overall, the five predictors retained in the final model (mean GTV, V5, D2cc [minimum dose to the hottest 2 cm³], circumferential length receiving ≥2.6 Gy/fx, and circumferential length receiving ≥3.0 Gy/fx) exhibited consistent selection across resamples, suggesting a degree of structural robustness. The remaining variables showed markedly lower inclusion frequencies, supporting the sparsity of the current final model. Complete frequencies are shown in **Figure 6**.

### 3.8 Sensitivity and robustness analysis

**Figure 7:**
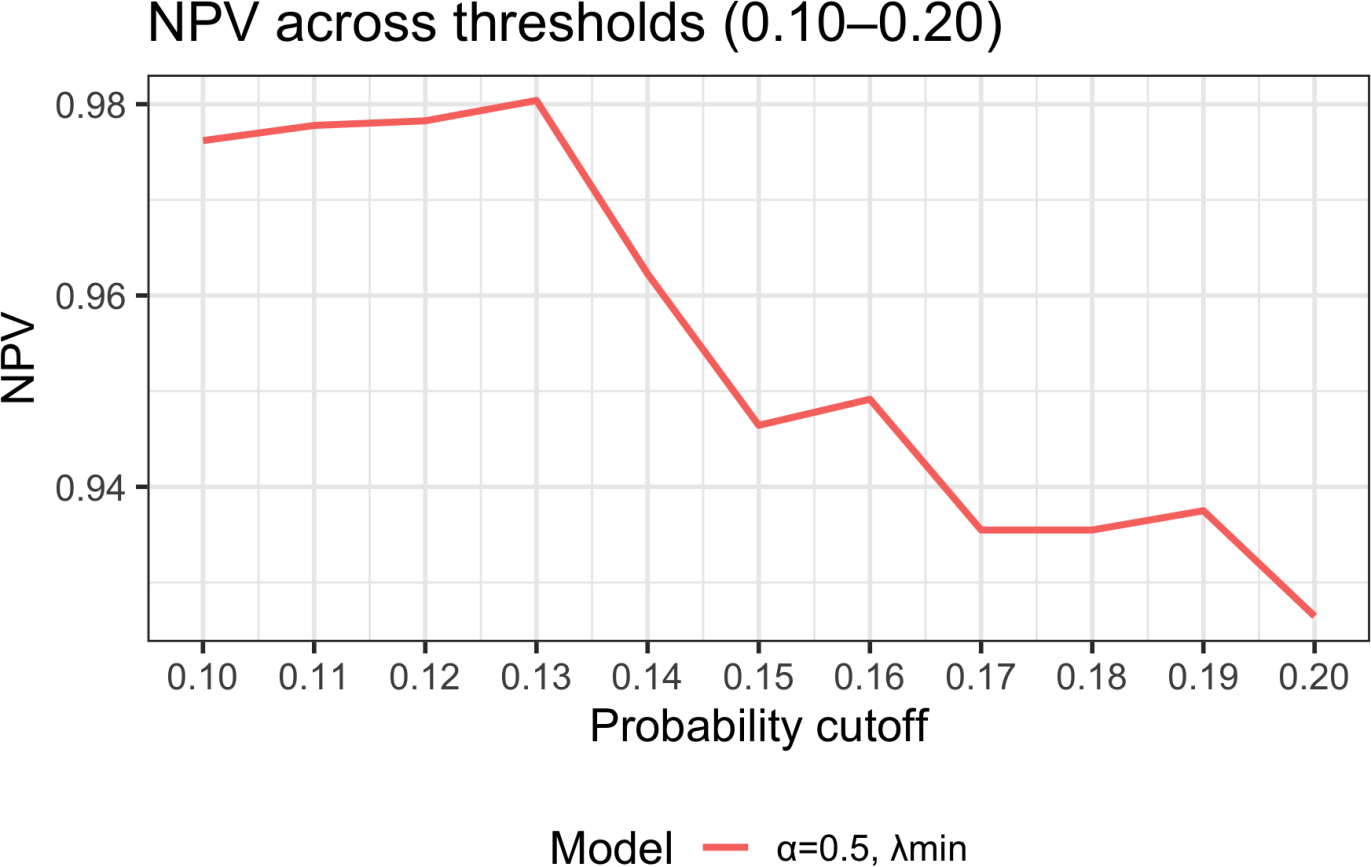
Threshold sensitivity analysis: NPV across probability thresholds (0.10–0.20). NPV–threshold curves derived from the final model (elastic-net α = 0.5, λ_min). NPV is stable at 97.5%–98.0% between 0.10 and 0.13, with a peak at 0.13 (~98.0%); at thresholds ≥0.15, NPV declines to 93.5%–94.6%. Together with the optimal cutoff 0.13 (sensitivity 94.1%, specificity 56.8%, PPV 29.6%, NPV 98.0%), the model demonstrates superior performance for ruling out ≥G3 RIE, enabling patients with predicted probability <0.13 to be classified as low risk for streamlined follow-up and resource allocation.

To assess the impact of threshold selection on clinical use, we plotted NPV as a function of the decision threshold within 0.10–0.20 (**Figure 7**). Between 0.10 and 0.13, NPV remained stable at 97.5%–98.0%, peaking at ~98.0% at 0.13. When the threshold rose to ≥0.15, NPV declined to ~93.5%–94.6%. Coupled with the Youden-based optimal cutoff of 0.13 (sensitivity 94.1%, specificity 56.8%, PPV 29.6%, NPV 98.0%), these findings indicate the model is better suited to “rule-out” applications: classifying patients with predicted probability <0.13 as low risk permits safe reduction of unnecessary interventions while optimizing follow-up and allocation of supportive care resources.

### 3.9 Nomogram visualization

**Figure 8:**
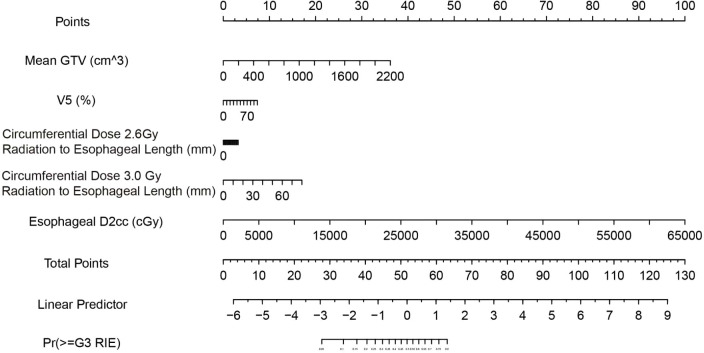
Nomogram for predicting ≥G3 RIE. Read each variable’s points from the “Points” axis, sum to obtain “Total Points,” and map to “Pr(≥G3 RIE)” for individualized risk. Units and variable labels are shown within the figure.

Based on the final model (elastic-net selection followed by Firth-penalized logistic regression), we constructed a nomogram (**Figure 8**). The five predictors included mean GTV, V5, D2cc, circumferential length receiving 2.6 Gy per fraction, and circumferential length receiving 3.0 Gy per fraction. Each predictor’s value is mapped to a points scale weighted by its regression coefficient; the summed points (Total Points) are then converted to the predicted probability of ≥G3 RIE.

Instructions for Clinical Workflow:

- Obtain the values of the five specified indicators during the treatment planning stage.
- Locate each value on the corresponding axis of the nomogram, record the points, and calculate the total score.
- Refer to the probability axis under “Total Points” to determine the individualized risk.
- Apply the recommended decision threshold: a predicted probability ≥0.13 denotes “high risk,” whereas <0.13 denotes “low risk.” At this threshold, the model demonstrated in this cohort a sensitivity of 94.1%, specificity of 56.8%, PPV of 29.6%, NPV of 98.0%, and overall accuracy of 62.9%, making it particularly suitable for clinical scenarios aimed at excluding patients at high risk.

## 4 Discussion

This study developed a prediction model for grade ≥3 RIE in lung cancer patients receiving MHRT, centering on dosimetric and geometric metrics that are directly amenable to modification during treatment planning. After variable selection by elastic-net regularization and model fitting with Firth-penalized logistic regression, five predictors were retained: mean GTV, V5, D2cc, and the circumferential irradiated lengths at 2.6 Gy and 3.0 Gy. Apparent discrimination was good (AUC=0.771); after correction via a fully nested bootstrap (B=1000), the AUC was 0.608 (95% CI, 0.464–0.761), and the Hosmer–Lemeshow test indicated acceptable overall fit (p = 0.449). At a probability threshold of 0.13, the model achieved a high NPV (98.0%), making it well suited to ruling out patients at risk of severe RIE. Decision-curve analysis showed positive net benefit across threshold probabilities of 0.05–0.60, supporting clinical utility within commonly accepted risk-tolerance ranges.

Most prior RIE modeling has focused on conventional fractionated radiotherapy (CFRT), typically relying on univariable or standard multivariable logistic regression, with limited stability and predictive power. Kim de Ruyck et al. noted that single dosimetric metrics have modest predictive value, whereas integrating clinical characteristics, treatment parameters, and genetic information can raise the AUC to 0.87, outperforming single-factor models (14). Existing RIE risk models, often derived from CFRT or SBRT cohorts, generally depend on univariable screening or traditional multivariable selection and lack systematic control of overfitting. Our findings underscore the joint importance of high-dose hotspots (D2cc) and low-dose spread (V5), and further quantify circumferential dose coverage, thereby enriching dose–response evidence in the context of moderate hypofractionation. Although the optimism-corrected AUC fell to the moderate range, it is comparable to internally validated models developed under small-event, small-sample conditions. Given the ≈16% incidence of ≥G3 RIE, the model’s high NPV confers particular value for prognostic stratification.

In recent years, MHRT has been increasingly adopted in lung cancer because it shortens treatment courses and increases BED, making it especially suitable for patients with poorer baseline status or those receiving sequential chemoradiotherapy (7, 15). This benefit, however, is accompanied by higher rates of acute radiation toxicity; prior studies have shown that MHRT is more likely than CFRT to induce severe RIE (16). By focusing on this specific population, our work addresses a gap in risk prediction for RIE under moderate hypofractionation and thus holds meaningful clinical relevance.

In practice, the model can support individualized risk assessment at the pre-treatment planning stage. Clinicians may estimate a patient’s risk using the nomogram or the accompanying R script based on the five key variables. Using the 0.13 threshold, many patients can be classified as low risk (predicted probability < 0.13), corresponding to a low likelihood of ≥G3 RIE (NPV ≈ 98%), which facilitates routine follow-up and allows resources to be focused on higher-risk individuals. For patients exceeding the threshold, one may consider: ①reducing D2cc and shortening or limiting the circumferential length exposed to high per-fraction doses at the planning stage; ② early intensification of nutrition, analgesia, and mucosal protection; and ③ shortening reassessment intervals to enable timely intervention, thereby lowering the incidence of severe adverse events and preserving adherence and efficacy (17).

With respect to individual variables, mean GTV emerged as a major risk factor for RIE. Larger tumors typically necessitate broader target volumes, increasing the cumulative dose to the esophagus and adjacent tissues. In addition, larger volumes often entail more complex planning with less homogeneous dose distributions and an increased likelihood of local hotspots, exacerbating radiation injury to the esophageal mucosa. Werner-Wasik et al. observed that expanding fields involve more mediastinal normal tissue and elevate esophageal cumulative dose, thereby increasing the risk of severe esophagitis (18). Wei et al. likewise reported that greater tumor burden significantly heightened acute esophageal toxicity during concurrent chemoradiotherapy (19). More direct evidence comes from Monti et al. (2022), who showed that larger GTVs expand esophageal high-dose regions (e.g., D2cc, Dmax), with a corresponding rise in RIE risk (20). Together, these findings support our conclusion that tumor volume is an indispensable predictor in RIE risk assessment.

V5—the proportion of esophageal volume receiving ≥5 Gy—captures the extent of low-dose spread and was strongly associated with severe RIE. Traditional assessments have emphasized mid-to-high dose metrics (e.g., V50, Dmean), but recent work suggests that extensive low-dose exposure can meaningfully contribute to acute esophagitis. Radiobiological studies indicate that large-volume, low-dose irradiation can induce chronic low-grade inflammation, endothelial injury, extracellular-matrix remodeling, and impaired repair, thereby aggravating radiation damage (21). Kaymak Cerkesli et al. found that higher V5 correlated with grade 1–2 esophagitis and with weight loss and hypoalbuminemia, implying that chronic inflammation may blunt mucosal healing (22). In patients with locally advanced NSCLC, Paximadis et al. similarly emphasized that the cumulative burden of low-dose regions is a non-negligible driver of acute toxicity, underscoring the need to optimize low-dose distributions during clinical planning (23).

D2cc denotes the minimum dose delivered to the hottest 2 cm³ of esophageal tissue—an index of localized peak exposure—and is highly sensitive to the risk of tissue injury. Given the marked dose sensitivity of the esophageal mucosa, this parameter possesses substantial predictive value. Elevated D2cc typically signifies the presence of focal “hotspot” regions capable of triggering acute mucosal necrosis, deep ulceration, and a self-propagating inflammatory cascade, ultimately resulting in significant functional impairment. Herr et al. likewise supported D2cc as a key dosimetric predictor of RIE and emphasized prioritizing its control during planning, particularly in high-risk patients, to curb the incidence of severe esophagitis (23). In a separate multicenter prospective study of NSCLC patients undergoing radiotherapy, Herr et al. found that D2cc was significantly associated with both grade ≥2 and grade ≥3 acute esophagitis: at D2cc = 61 Gy, the risk of grade ≥3 esophagitis was 3%; among patients receiving concurrent chemoradiotherapy, D2cc = 50 Gy corresponded to a 50% risk of grade ≥2 esophagitis (10). Thus, as a proxy for localized high-dose exposure, rising D2cc is tightly linked to RIE risk. Accordingly, beyond controlling mean dose, planning should explicitly mitigate hotspots—via multi-objective optimization or hotspot-penalty strategies—to reduce severe RIE and enhance treatment safety and quality of life.

Conceptually, D2cc captures focal hotspot exposure that can breach the mucosal barrier and ignite a local inflammatory cascade, whereas V5 reflects diffuse low-dose accumulation—the “background noise” of subclinical inflammation and reparative burden. Larger GTV and longer circumferential dose-coverage lengths indicate greater irradiated volume/length and tighter field wrap around the esophagus, consistent with heightened mucosal-injury risk. The coherent directions of effect within the same model justify these factors as actionable levers for plan optimization.

Beyond conventional DVH metrics such as V5 and D2cc, our model also selected circumferential irradiated lengths at 2.6 Gy and 3.0 Gy, indicating that not only overall dose level but also the axial and circumferential dose distribution along the esophagus materially influences RIE risk. The L-dose (length–dose) construct has been proposed to systematically evaluate the relationship between irradiated length at varying dose levels and clinical toxicity. Higher L-dose has been associated with significant weight loss, indirectly reflecting the detrimental impact of long-segment, low-to-intermediate dose exposure on mucosal integrity and function—findings that accord with our observation that circumferential dose-coverage length tracks with RIE risk (24). Moreover, leveraging real-world data and validation in the PORT-C randomized trial, Ma et al. demonstrated that dosiomic models incorporating spatial dose features (e.g., local high-dose clustering, field morphology) outperform traditional DVH parameters for predicting RIE, underscoring the mechanistic importance of spatial dose patterns. This aligns with our results and suggests that jointly assessing “irradiated extent” and “local dose concentration” may surpass single-volume metrics in forecasting acute esophagitis (25).

Specifically, we observed that as the esophageal length receiving 2.6 Gy and 3.0 Gy increases, the risk of RIE rises appreciably. Given the esophagus’s moderate tolerance, long-segment low-dose exposure can disrupt mucosal continuity, delay epithelial repair, and sustain inflammation and fibrosis, thereby intensifying acute toxicity. Notably, 2.8 Gy was not retained in the final model, suggesting a potentially non-linear dose–length effect across low-dose levels—an observation that merits confirmation in larger cohorts to clarify threshold behavior.

At the Youden-optimal threshold of 0.13, overall accuracy was 62.9%; sensitivity, 94.1%; specificity, 56.8%; NPV, 98.0%; and PPV, 29.6%. The high NPV indicates strong rule-out capacity for non-severe RIE, potentially reducing unnecessary pre-treatment interventions, while the high sensitivity favors comprehensive capture of high-risk individuals to support risk stratification and clinical decision-making. Decision-curve analysis showed positive net benefit across commonly used thresholds (~0.05–0.60). The nomogram is concise and intuitive, enabling rapid estimation of an individual’s probability of grade ≥3 RIE from five key dosimetric/geometric factors, thereby facilitating plan optimization and clinician–patient communication.

These findings should be interpreted in the context of the study design and cohort composition. We centered the analysis on planning-stage, directly modifiable dosimetric/geometric metrics; combined elastic-net selection with Firth correction; and employed fully nested bootstrap validation to temper overfitting and yield conservative performance estimates under limited event counts. Conversely, the single-center, retrospective dataset and the small number of events imply that generalizability is greatest to similar populations and workflows; although DVH-based and circumferential-length measures are actionable, their values may vary with contouring practices and planning strategies. The 0.13 decision threshold reflects a rule-out orientation suited to our data and clinical trade-offs; in settings with different prevalences or resource constraints, lightweight recalibration (intercept/slope) is advisable before deployment. With multicenter external validation and the integration of multimodal information (e.g., inflammatory biomarkers, radiomics), calibration and generalizability should improve further, preserving clinical operability while enhancing discrimination and robustness.

## 5 Conclusion

Leveraging dosimetric/geometric information that is directly modifiable at the planning stage, we developed and internally validated a parsimonious model to predict ≥G3 RIE after MHRT in lung cancer. The final model comprises five key factors—mean GTV, V5, D2cc, and the circumferential lengths at 2.6 Gy/fraction and 3.0 Gy/fraction—and maintained moderate discrimination and acceptable calibration after fully nested bootstrap correction. At a decision threshold of 0.13, the model provides a high NPV, favoring rule-out use and upstream stratification during treatment planning. The accompanying nomogram affords a bedside-friendly tool for rapid estimation and communication, and may support plan optimization (e.g., lowering D2cc, shortening high-per-fraction circumferential coverage) and peri-treatment supportive interventions.

Given the single-center retrospective design and limited events, broader deployment will require multicenter external validation and light recalibration (intercept/slope). Future work integrating radiomics/inflammatory biomarkers and prospective impact evaluation—assessing reductions in severe RIE and treatment interruptions—may further enhance discriminative performance and stability while retaining clinical practicality.

## Data Availability

The raw data supporting the conclusions of this article will be made available by the authors, without undue reservation.
