# Supplementary material for: Development and validation of a predictive model for severe radiation-induced esophagitis in lung cancer patients undergoing moderate hypofractionated radiotherapy
Source: Front Oncol. 2025 Oct 3;15:1656907. doi: 10.3389/fonc.2025.1656907 (PMC12531044; doi:10.3389/fonc.2025.1656907)
Supplement: Supplementary file 1 [file DataSheet1.docx]

Supplementary Material

# Supplementary Figures and Tables

## Supplementary Figures


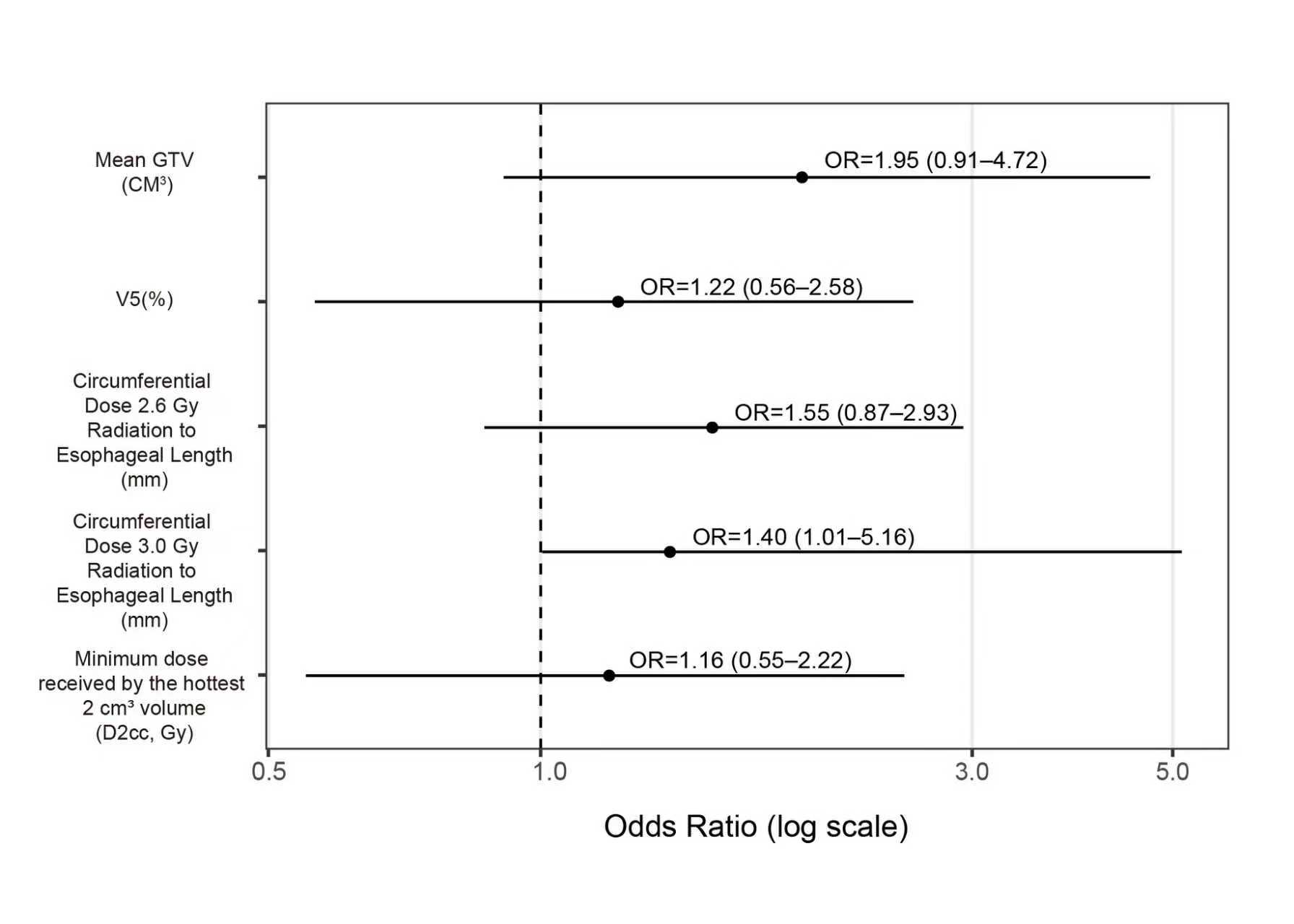


**Figure 1.** Forest plot for predicting ≥G3 RIE (Firth-penalized logistic regression)

Black dots denote OR point estimates, horizontal lines the 95% CIs; the vertical dashed line marks OR = 1 (no effect); the x-axis is on a logarithmic scale. Because continuous predictors were z-standardized before model entry, ORs represent the change in risk per one–standard deviation increase.

## Supplementary Figures


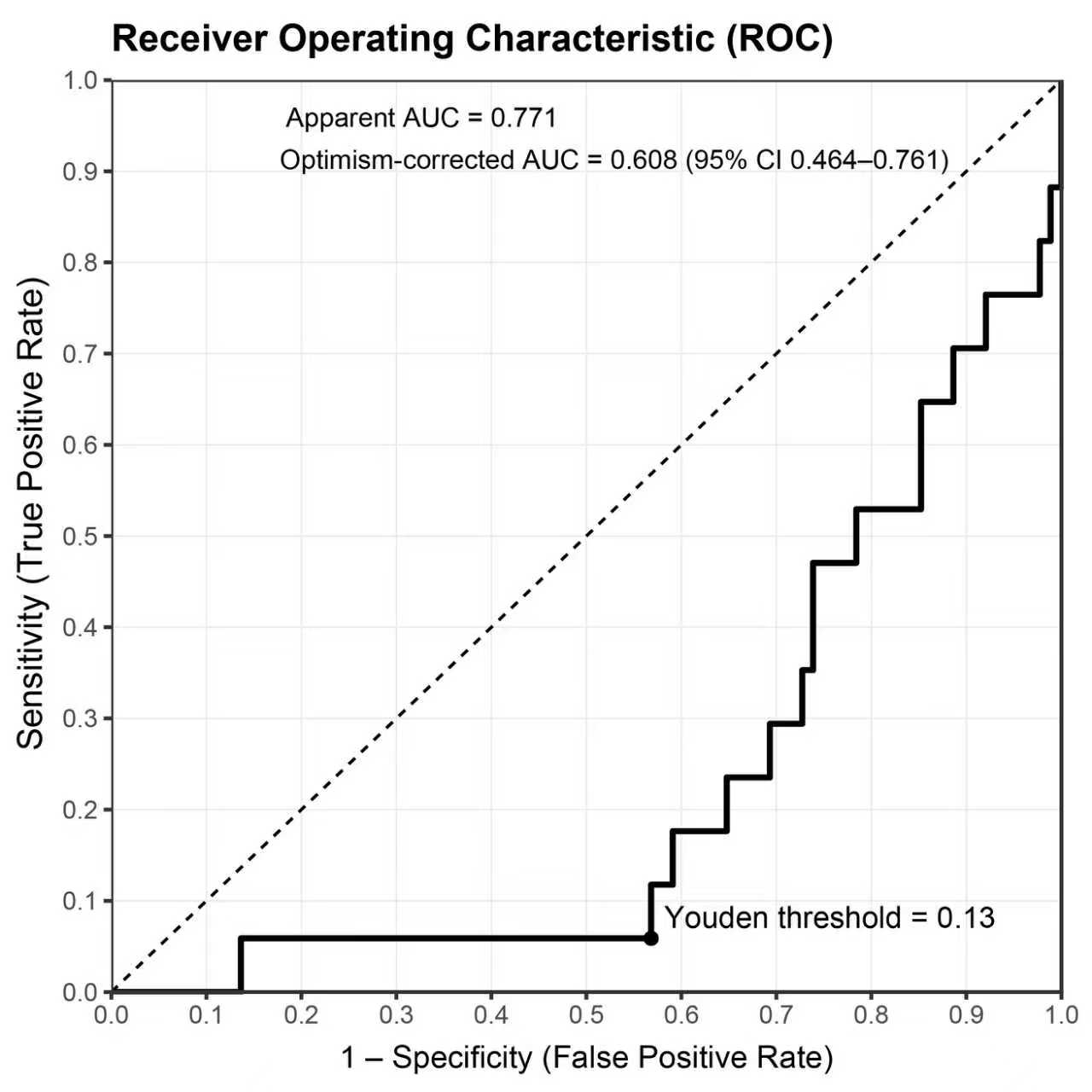


**Figure 2.** ROC curve and threshold performance for predicting ≥G3 RIE

The bold black line depicts the apparent ROC (AUC = 0.771); the dashed diagonal represents chance. After fully nested bootstrap correction, AUC = 0.608 (95% CI 0.464–0.761). The solid dot marks the optimal cutoff based on the maximal Youden index (0.1299 ≈ 0.13). The x- and y-axes denote 1 − specificity (false-positive rate) and sensitivity (true-positive rate), respectively. Sample size N = 105; events = 17 (16.2%). Predicted probabilities derive from the full-sample model using elastic-net selection followed by Firth-penalized logistic regression; continuous predictors were z-standardized prior to modeling.

## Supplementary Figures


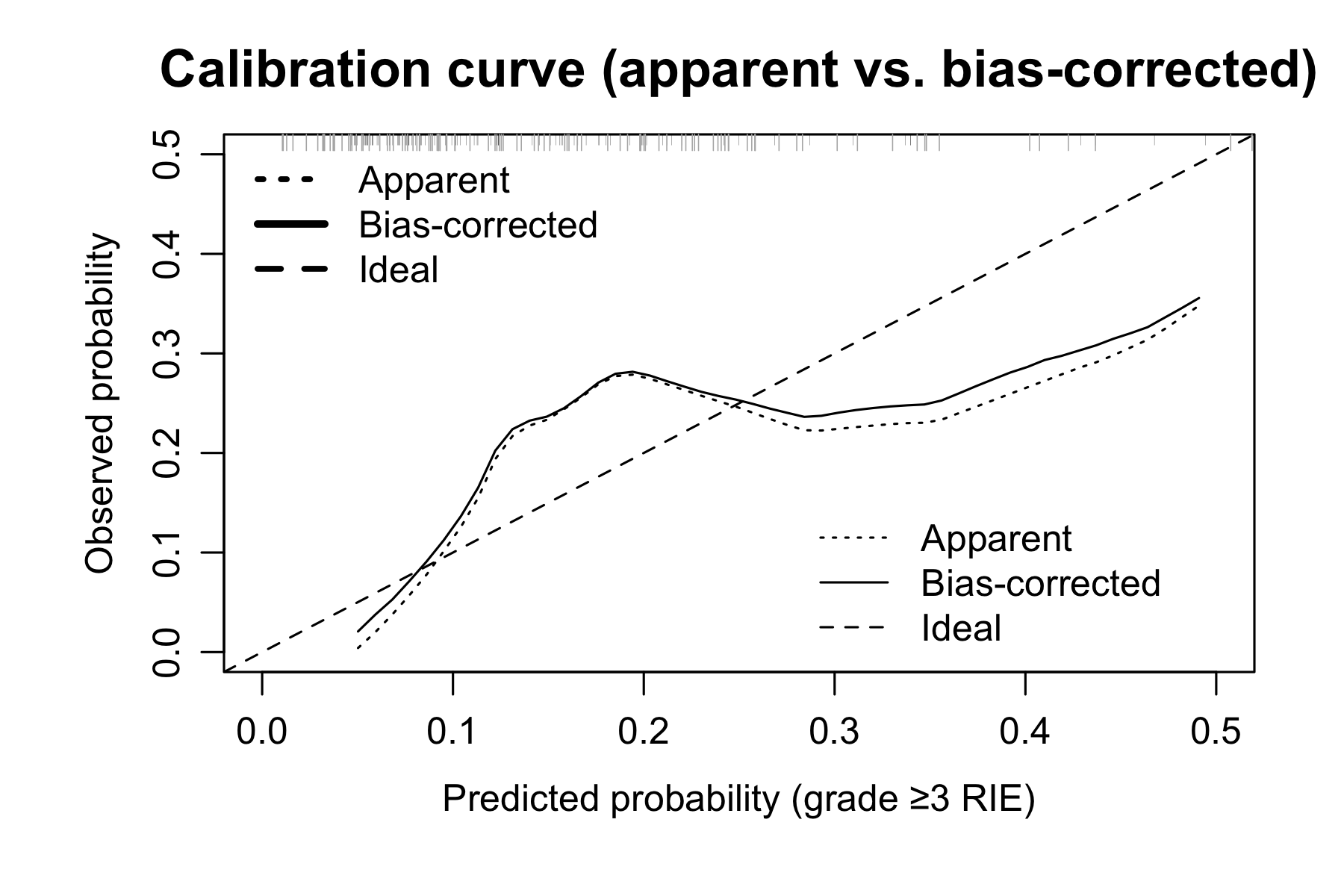


**Figure 3.** Calibration curves (apparent vs bias-corrected)

The dotted line denotes apparent calibration, the solid line the bootstrap bias-corrected curve (B= 1000), and the dashed line the ideal y = x; rug marks above the axis indicate the distribution of predicted probabilities. Alignment with the ideal line in the low–moderate range (~0.10–0.25) supports overall acceptable calibration. Apparent slope/intercept were 1.16/0.13; Hosmer–Lemeshow χ² = 7.84, p = 0.449.

## Supplementary Figures


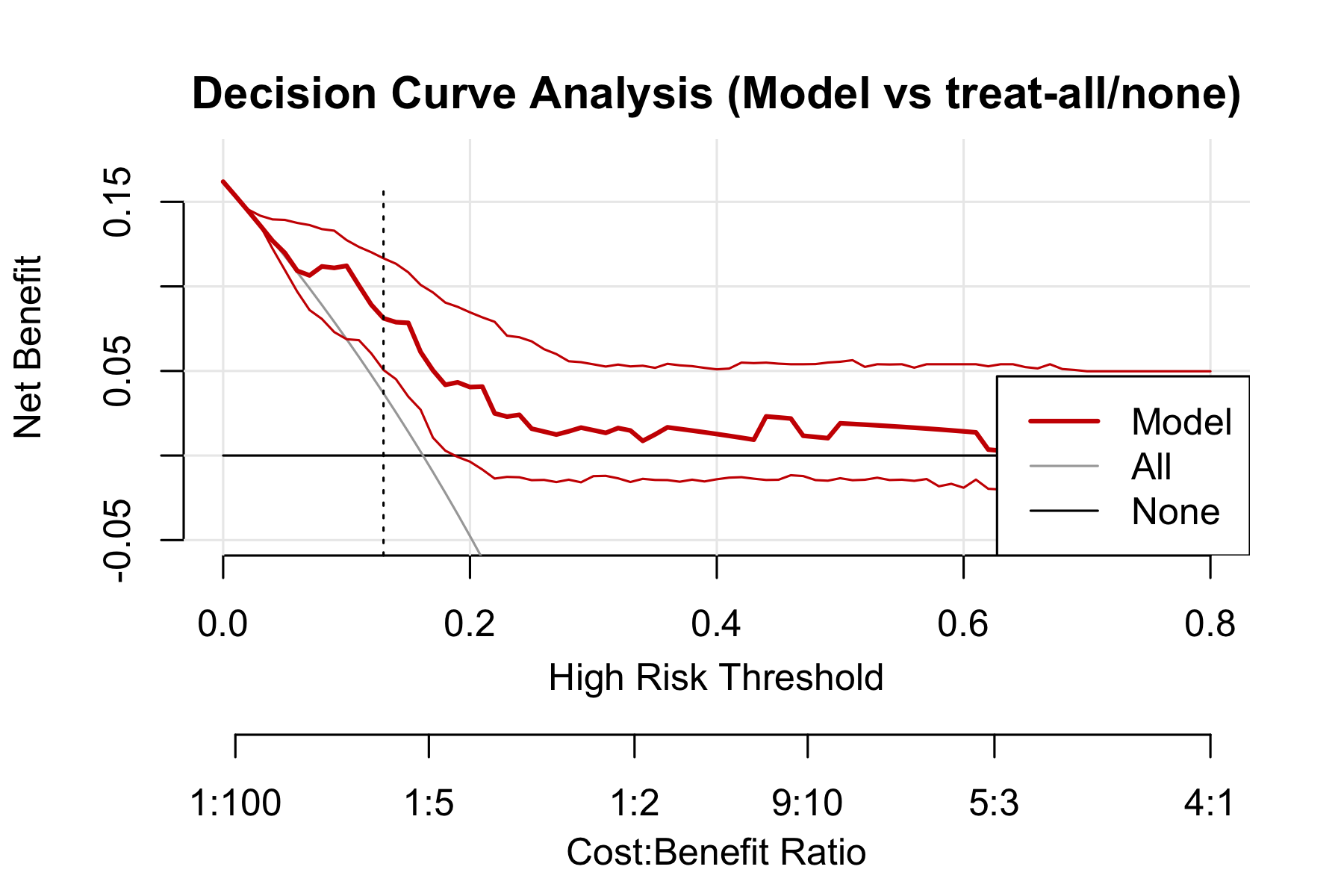


**Figure 4.** Decision Curve Analysis

The red solid line denotes model net benefit; the gray and black lines represent “treat-all” and “treat-none,” respectively. The vertical dashed line marks the Youden-based optimal threshold (pt ≈ 0.13). Net benefit is positive and exceeds both extreme strategies between 0.05 and 0.35, tapering toward the baselines at higher thresholds.

## Supplementary Figures


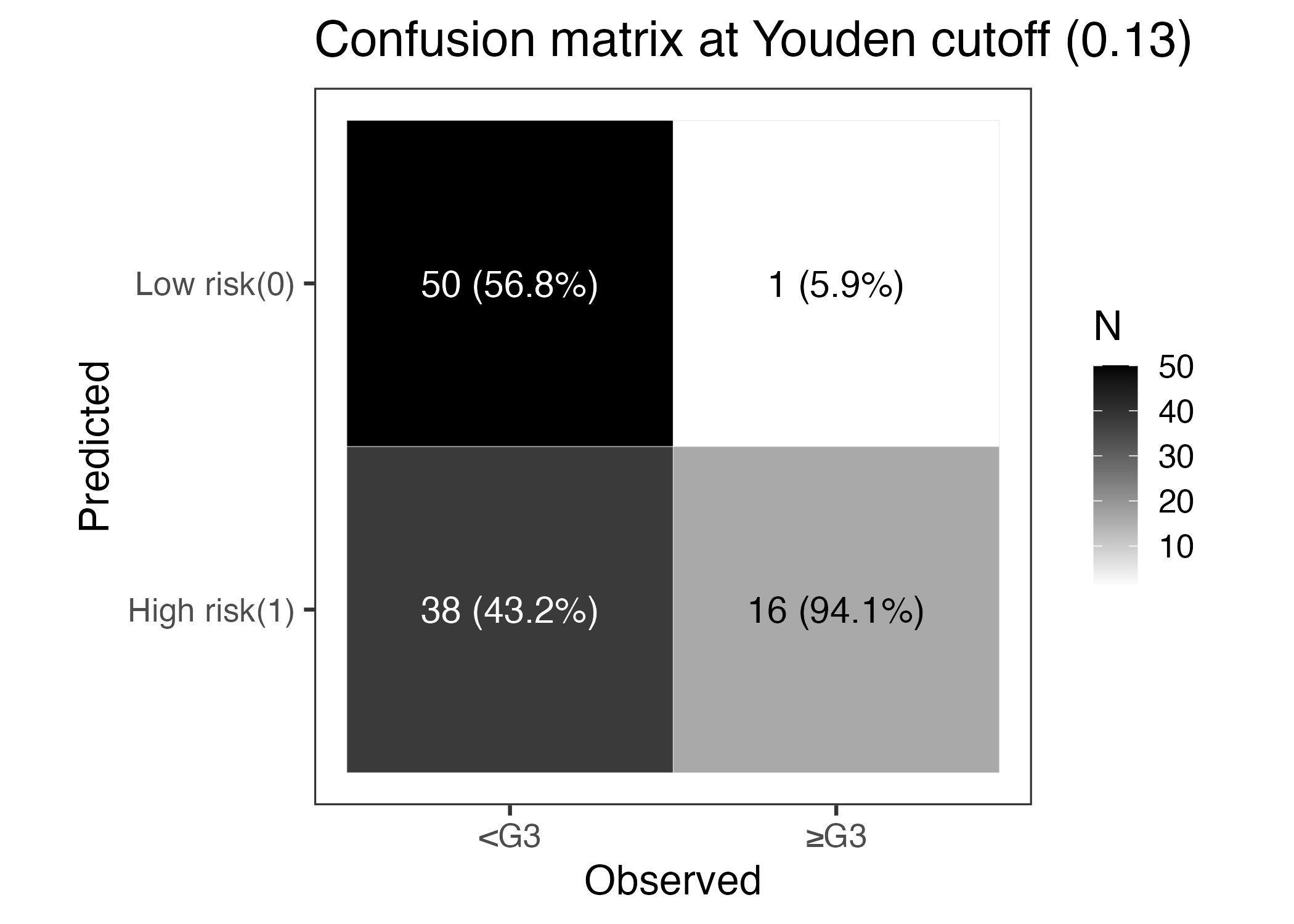


**Figure 5.** Confusion matrix at the Youden threshold (pt = 0.13)

Rows indicate model predictions (top: low risk [0]; bottom: high risk [1]); columns indicate observed outcomes (left: no ≥G3 RIE; right: ≥G3 RIE). Each cell shows counts, with column percentages in parentheses (proportion within the observed-outcome column): TN = 50 (56.8%), FN = 1 (5.9%), FP = 38 (43.2%), TP = 16 (94.1%). Derived metrics: sensitivity 94.1%, specificity 56.8%, accuracy 62.9%, PPV 29.6%, NPV 98.0%. The grayscale bar on the right encodes count magnitude.

## Supplementary Figures


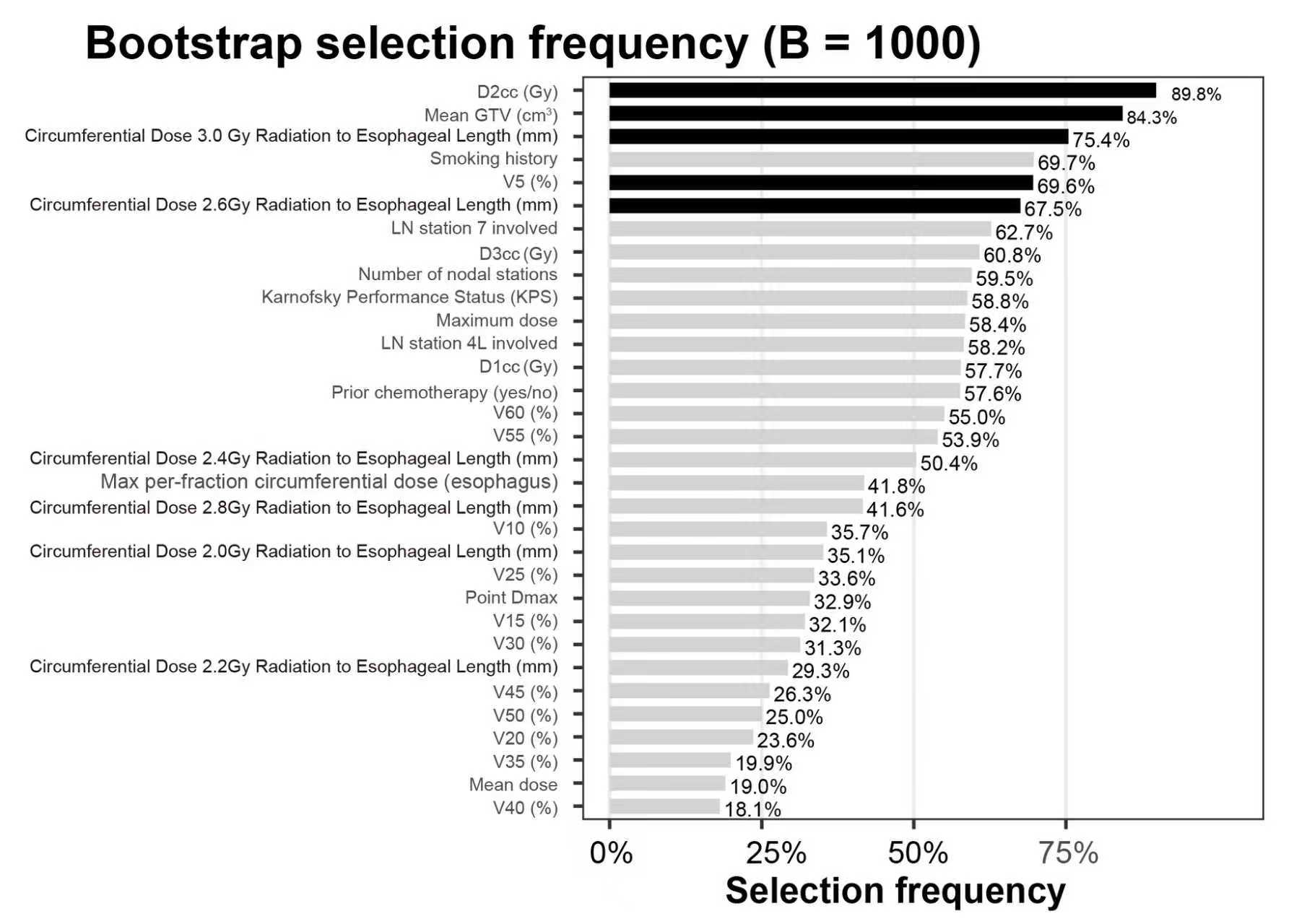


**Figure 6.** Variable selection frequencies (B=1,000)

The bar chart depicts, for each candidate, the proportion selected into the model across 1,000 fully nested bootstrap iterations (selection frequency, %). Black bars denote the five retained predictors: mean GTV (cm³), V5 (%), circumferential length ≥2.6 Gy/fx (mm), circumferential length ≥3.0 Gy/fx (mm), and D2cc³ (Gy). Gray bars indicate non-selected candidates. Values above bars represent selection frequency percentages.

## Supplementary Figures


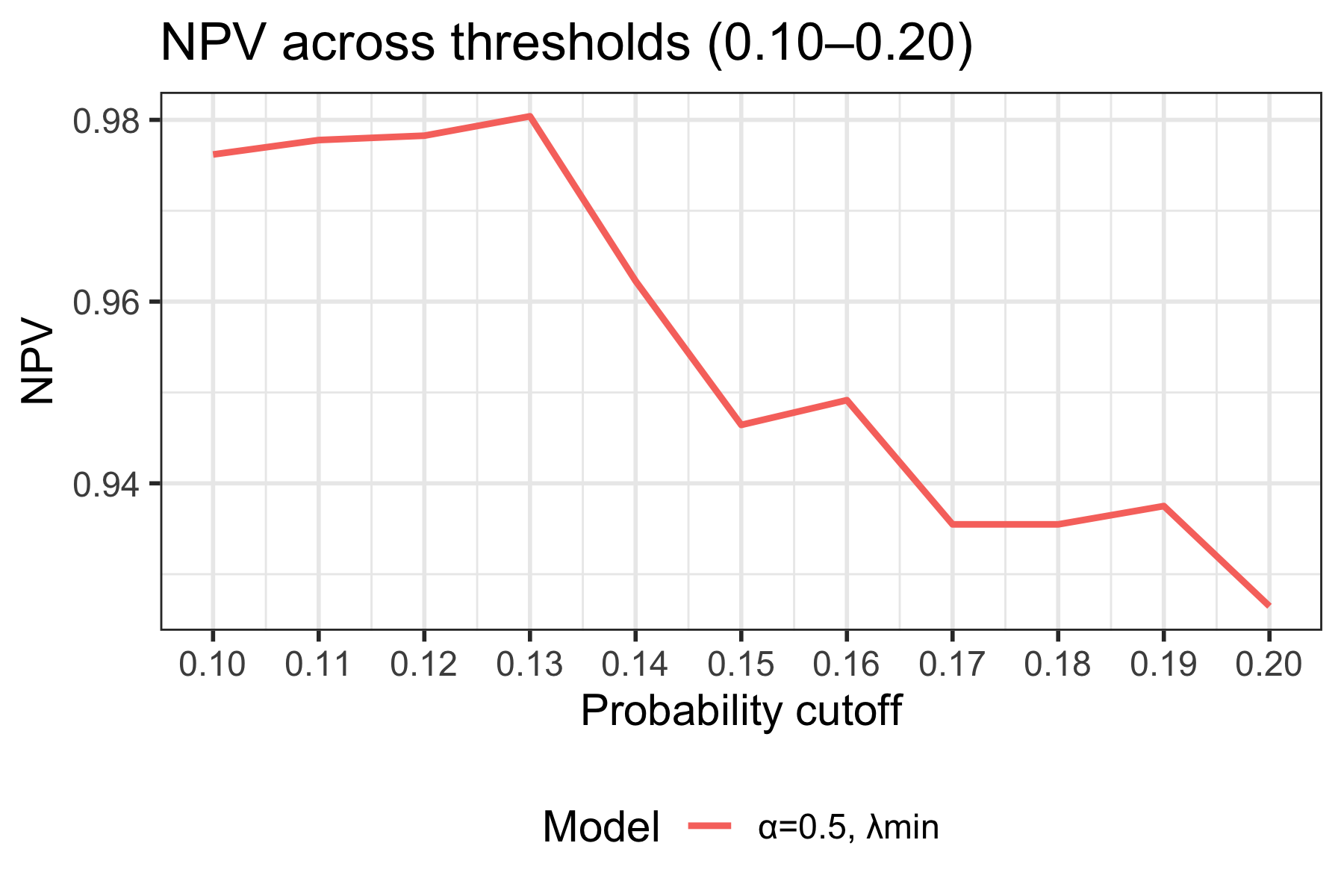


**Figure 7.** Threshold sensitivity analysis: NPV across probability thresholds (0.10–0.20)

NPV–threshold curves derived from the final model (elastic-net α = 0.5, λ_min). NPV is stable at 97.5%–98.0% between 0.10 and 0.13, with a peak at 0.13 (~98.0%); at thresholds ≥0.15, NPV declines to 93.5%–94.6%. Together with the optimal cutoff 0.13 (sensitivity 94.1%, specificity 56.8%, PPV 29.6%, NPV 98.0%), the model demonstrates superior performance for ruling out ≥G3 RIE, enabling patients with predicted probability <0.13 to be classified as low risk for streamlined follow-up and resource allocation.

## Supplementary Figures


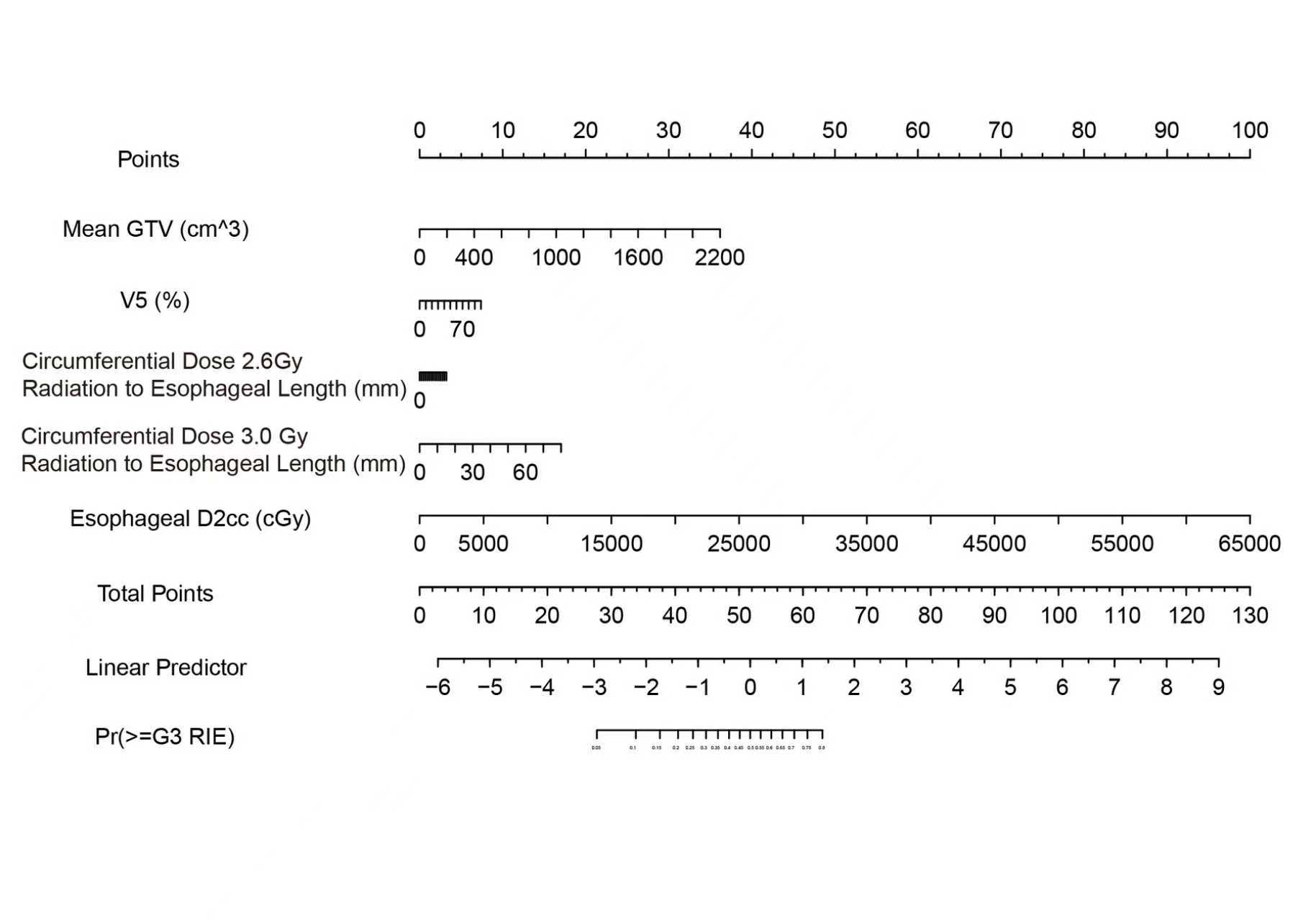


**Figure 8.** Nomogram for predicting ≥G3 RIE

Read each variable’s points from the “Points” axis, sum to obtain “Total Points,” and map to “Pr(≥G3 RIE)” for individualized risk. Units and variable labels are shown within the figure.
